# Tackling Histoplasmosis Infection in People Living with HIV from Latin America: From Diagnostic Strategy to Public Health Solutions

**DOI:** 10.3390/jof9050558

**Published:** 2023-05-11

**Authors:** Diego H. Cáceres, Beatriz L. Gómez, Ángela M. Tobón, Ángela Restrepo, Tom Chiller, Mark D. Lindsley, Jacques F. Meis, Paul E. Verweij

**Affiliations:** 1 Center of Expertise in Mycology Radboudumc/Canisius Wilhelmina Hospital, 6532 SZ Nijmegen, The Netherlands; jacques.meis@gmail.com (J.F.M.); paul.verweij@radboudumc.nl (P.E.V.); 2 Studies in Translational Microbiology and Emerging Diseases (MICROS) Research Group, School of Medicine and Health Sciences, Universidad del Rosario, Bogota 111221, Colombia; beatrizlgomez@hotmail.com; 3 IMMY, Norman, OK 73069, USA; 4 Instituto Colombiano de Medicina Tropical, Universidad CES, Medellín 055450, Colombia; angelamtobon@hotmail.com; 5 COLCIENCIAS Emeritus Researcher, Ministerio de Ciencias, Tecnología e Innovación, Bogota 111321, Colombia; angelares@une.net.co; 6 Mycotic Diseases Branch, Centers for Disease Control and Prevention (CDC), Atlanta, GA 30329, USA; tnc3@cdc.gov (T.C.); mil6@cdc.gov (M.D.L.); 7 Department I of Internal Medicine, Excellence Center for Medical Mycology, University Hospital Cologne, 50931 Cologne, Germany

**Keywords:** *Histoplasma*, histoplasmosis, VIH, Latin America, diagnostic, public health

## Abstract

Histoplasmosis, caused by the thermally dimorphic fungus *Histoplasma* spp., is a disease with a broad clinical spectrum, presenting from asymptomatic/flu-like symptoms to progressive disseminated disease in people with immunosuppression. In recent years, the concept of histoplasmosis as a disease restricted to the American continent has changed, as now histoplasmosis is reported in many regions around the world. In Latin America, histoplasmosis represents a threat, especially in people with advanced HIV disease (AHD). Diagnosis of histoplasmosis in people living with HIV (PLHIV) is challenging due to the low index of suspicion of the disease, non-specificity of signs and symptoms, and limited access to specific laboratory testing, while the diagnostic delay is significantly associated with mortality. In the last decade, novel diagnostic tests have been developed for the rapid detection of histoplasmosis, such as commercial kits for antigen detection. Furthermore, advocacy groups were created that presented histoplasmosis as a public health problem, with emphasis on patients at risk of progressive disseminated disease. This review aims to discuss the impact of histoplasmosis associated with AHD in Latin America and the strategies employed to tackle histoplasmosis, from the implementation of laboratory testing to disease advocacy and public health interventions.

## 1. *Histoplasma* spp.

The *Histoplasma* genus is composed of two species: *H. capsulatum* and *H. farciminosum*. *H. capsulatum* has two varieties associated with disease in humans: *H. capsulatum* var. *capsulatum*, which is globally distributed, and *H. capsulatum* var. *duboisii*, which is restricted to the African continent [1,2]. The second species, *H. farciminosum*, is recognized for causing disease in horses in the African continent [1,2]. In recent years, with evolving taxonomic insights, the reclassification of *H. capsulatum* has been proposed. The most widely used classification by Kasuga et al. [3] is based on multi-locus sequence typing (MLST). It divides *H. capsulatum* into eight clades, including two clades in North America: class 1 clade and class 2 clade (clades i and ii); two Latin American clades, group A clade and group B clade (clades iii and iv); an Australian clade (clade v); the Netherlands (Indonesian?) clade (clade vi); the Eurasian clade (vii); and the African clade (viii). Seven of eight clades represent groups that may be recognized as phylogenetic species. The African clade includes all *H. capsulatum* var. *duboisii* individuals [3]. More recently, Sepulveda et al. proposed dividing *H. capsulatum* var. *capsulatum* into four species using whole-genome sequencing. Based on the results, the authors proposed the following species: (i) *Histoplasma capsulatum sensu stricto Darling 1906*, (ii) *Histoplasma mississippiense sp*. *nov.*, (iii) *Histoplasma ohiense sp. nov.*, and (iv) *Histoplasma suramericanum sp. nov*. [4]. It is important to mention that at the time of writing this review, these reclassifications have not been validated, and approval of this new classification is still in discussion [5].

## 2. Histoplasmosis

Histoplasmosis is acquired when the host is exposed to an environment that contains the infective particles of the fungus, micro-conidia, and fragments of hyphae [6]. Infection can be acquired by inhalation of these infective particles due to disruption of contaminated soil that is enriched with bird and bat guano or as a result of work or recreational activities [7]. Histoplasmosis is not transmitted from person to person, although transmission through organ transplantation has been reported rarely [8,9]. After the inhalation of infective particles, a primary lung infection may develop [6]. Subsequently, the fungus may spread from the lung to other organs of the reticuloendothelial system by hematogenous dissemination [6]. More than 95% of infections resolve spontaneously, primarily in hosts without alteration of the immunological response and those exposed to a low burden of infective particles [6,10,11]. However, in most patients, the fungus is not eliminated, and the fungus then persists for many years in a latent state and is able to reactivate [6,10]. The clinical manifestations of histoplasmosis depend on various factors such as the inoculum inhaled during the environmental exposure, lung integrity, or immunological alterations present in the patient at the time of exposure [10,11]. These factors were considered by Goodwin et al., who proposed a classification for histoplasmosis in 1981 [11]. This classification aimed to combine epidemiological and clinical characteristics to classify disease stages and support treatment choice.

The first reported cases of histoplasmosis were identified in the early 20th century in Panama by the American pathologist Samuel Taylor Darling, during the time he worked at the Panama Canal. The first case in 1905 was a 27-year-old male from Martinique who worked on the Panama Canal construction and developed a fulminant disseminated disease. Darling observed in this patient that the lungs were studded with granulomas, resembling miliary tuberculosis. He also found granulomas in the spleen, liver, and bone marrow. Surprisingly, in 1906 a second Panama Canal worker who was also from Martinique presented with the same disseminated disease. The third case was in a Chinese immigrant who had been a resident in Panama for 15 years and showed similar microorganisms in histopathological analysis. Initially, Darling classified this disease as a protozoal infection, but in 1912, the disease was reclassified from a protozoal to a fungal disease by Henrique da Rocha-Lima, a pathologist and infectious disease physician from Brazil, who based this reclassification on a comparison of *Leishmania* and *Histoplasma* [12,13,14,15]. In 1933, William A. DeMonbreun isolated *Histoplasma* from a clinical human specimen for the first time [14,15], and in 1948, Chester W. Emmons isolated *Histoplasma* from soil samples [14,15]. In the early 1950s investigators from Suriname isolated *Histoplasma* from human, animal, and environmental samples [16,17,18]. These early observations in the 1950s from Suriname and Guyana in humans and animals did not generate attention; consequently, in the early 1960s, many Dutch military personnel stationed in Suriname were wrongly diagnosed with tuberculosis, but in retrospect had histoplasmosis [19,20,21].

In the 1960s, Leo Kaufman and the staff of the US Centers for Disease Control and Prevention (CDC) Mycotic Diseases Branch standardized the first immunodiagnostics assays for anti-*Histoplasma* antibodies (Ab) detection [22]. In 1986, Lawrence J. Wheat developed the first assay for *Histoplasma* antigen (Ag) detection in urine and serum, and in 2002 Ralf Bialek developed the first PCR protocols for detection of *Histoplasma* DNA in human tissues [23,24]. Before the 1980s, sporadic reports from Latin American countries described histoplasmosis cases, even though multiple seroprevalence studies using intradermal reactivity with histoplasmin were conducted in the region, demonstrating high rates of skin reaction in the general population [25,26]. This changed after the beginning of the HIV pandemic.

Since the first report of histoplasmosis in Panama in 1906, this disease has been described worldwide, with the highest frequency in the American continent. However, in recent years, histoplasmosis has evolved from a geographically restricted endemic mycosis to a global disease, with cases increasingly described in Africa and Asia. This increasing trend may be partly the result of greater disease awareness and the development and use of more accurate diagnostic assays [13,27].

## 3. Histoplasmosis and HIV

The term progressive disseminated histoplasmosis (PDH) refers to one of the clinical forms of histoplasmosis characterized by the inability of the host immune system to control the fungal infection [28,29]. In immunosuppressed hosts, histoplasmosis can be the result of a primary infection or a reactivation of a prior infection. Reactivation may occur in individuals who reside or were residents in regions where the fungus is in the environment [6,28,29]. Following the start of the AIDS epidemic, several patients with AIDS in the Netherlands, who originated from Suriname, presented with disseminated histoplasmosis, which was not surprising considering the previously described presence of *Histoplasma* in Suriname [30]. This pattern has been seen in many Latin American areas since 1987, where more than 90% of reported cases of PDH have occurred in patients with advanced HIV disease, leading the CDC to recognize this disease as an HIV opportunistic infection [31].

Since 2017, the World Health Organization (WHO) had released recommendations for rapid initiation of antiretroviral treatment (ART) in all people living with HIV (PLHIV) [32]. PLHIV with advanced disease are at elevated risk of developing histoplasmosis, especially those living in regions endemic for this disease [33]. Clinical signs and symptoms of histoplasmosis are non-specific in these patients, and accurate diagnosis of histoplasmosis is difficult, as is differentiation from coinfections with other opportunistic pathogens, such as mycobacteria [34]. Co-infections in PLHIV with histoplasmosis have been reported in up to 50% of cases and reports of co-occurrence with mycobacterial disease have ranged from 2% to 35% in Latin American countries, underscoring the need for adequate laboratory diagnostics [35,36,37,38].

For the Latin America region, histoplasmosis is among the most prevalent opportunistic infections affecting PLHIV [39]. This disease has been associated with high mortality rates in different cohorts of PLHIV that develop disseminated histoplasmosis (up to 30% mortality) [40]. For 2012, the estimated burden of histoplasmosis in PLHIV in Latin America ranged from 6710 to 15,657 cases per year. In some countries the prevalence and mortality associated with histoplasmosis was equivalent or higher than that of TB [26]. Based on this estimation, authors have concluded that histoplasmosis is a neglected disease among opportunistic infections caused by fungal pathogens in PLHIV in the Americas and could be responsible for a significant proportion of annual PLHIV deaths [26]. In Guatemala, in 2017, of 1953 PLHIV who were evaluated in a prospective cohort, 317 (16%) patients were diagnosed with opportunistic infections. Of those, 36% had tuberculosis (TB), 31% had histoplasmosis, 19% had cryptococcosis, 4% had nontuberculous mycobacterial disease, and 10% presented with coinfections. Histoplasmosis was the most frequent defining manifestation of advanced HIV in this cohort [41].

Nevertheless, some progress has been made in recent years, including the publication of WHO/Pan American Health Organization (PAHO) guidelines for the diagnosis and management of disseminated histoplasmosis in PLHIV [42], the development, validation, and commercial availability of kits for rapid detection of *Histoplasma* antigen in human specimens [43,44], and the inclusion of these rapid assays in the second WHO model list of essential in vitro diagnostics [45]. WHO histoplasmosis guidelines also present recommendations for treatment, including the use of liposomal amphotericin for induction therapy in patients with severe or moderately severe disease. Guidelines for these patients recommend the use of itraconazole for 12 months for maintenance therapy; shorter therapy could be considered if the patient is clinically stable and if their immune status has improved. These guidelines also recommend the rapid initiation of ART and describe recommendations for the treatment of co-infection with TB.

In addition, based on public health importance, global burden, and existing knowledge gaps, *Histoplasma* spp. has been recently recognized as a WHO fungal priority pathogen [46]. These achievements will help to estimate the real burden of histoplasmosis in PLHIV.

## 4. Diagnosis of Histoplasmosis in PLHIV

Symptoms of PDH are often nonspecific, and among patients with advanced HIV disease they resemble those of other infectious diseases, especially TB, complicating diagnosis and treatment [26,34,38]. Delayed treatment of histoplasmosis is a major cause of mortality (approximately 30% in PLHIV) [39,40]. Conventional laboratory methods used for diagnosing histoplasmosis, such as culture and histopathology, pose many challenges, including the need for complex laboratory infrastructure and experienced staff with mycology training, a long turnaround time of up to several weeks, and variable assay sensitivity, 72% to 81% (Table 1) [47]. Diagnosis using conventional serologic methods is further complicated by a reduction in antibody test sensitivity (38–70%) when performed in immunocompromised individuals (Table 1) [47].

Detection of circulating antigen in urine or sera has been described as the best choice to diagnose PDH in PLHIV [42,47,48]. A systematic review and meta-analysis compared the accuracy of different diagnostic assays for the diagnosis of histoplasmosis in PLHIV [47]. In this meta-analysis, antigen detection assays in urine and sera showed the highest performance in detecting histoplasmosis in PLHIV (pooled sensitivity, 95%, and pooled specificity, 97%) (Table 1) [47]. Advantages of antigen assay include its commercial availability and the ability to be implemented in a laboratory with lower-level biosecurity (levels 1 and 2). In addition, antigens are present only during active disease, unlike antibodies, which are detected long after infection has been resolved. The review and meta-analysis also indicated that molecular testing may be a desirable alternative for correct diagnosis of histoplasmosis in PLHIV [47]. However, a lack of commercially available kits and standardized methods, and the limited number of validation studies, limit their broad use in clinical microbiology laboratories [47]. It has yet to be determined which DNA extraction method, gene target and primers, or amplification methodology is optimal; it will likely vary for culture confirmation and direct detection in patient specimens [47]. Furthermore, the 2020 EORTC/MSGERC definitions of invasive fungal diseases do not include molecular detection assays as methods for the diagnosis of endemic mycoses due to a lack of consensus on gene targets and laboratory protocols for molecular assays [49]. Further investigations, laboratory consensus, and development of commercial kits are needed.

## 5. Histoplasmosis in PLHIV in Latin America

A literature review was conducted using PubMed Central and LILACS. The searches were limited to those studies published in English, Spanish, and Portuguese, and used as search terms histoplasmosis AND HIV and terms including their synonyms. We included case series or cohort studies from Latin American countries that described a minimum of ten histoplasmosis cases. Studies were excluded if they were not focused on human or environmental studies or if they constituted literature reviews. A total of 34 papers describing cases series or cohort studies involving PLHIV with histoplasmosis were identified [37,38,50,51,52,53,54,55,56,57,58,59,60,61,62,63,64,65,66,67,68,69,70,71,72,73,74,75,76,77,78,79,80]. These papers describe cases from Central American countries, including El Salvador, Guatemala, Honduras, Nicaragua, and Panama, and South American countries, including Argentina, Brazil, Colombia, French Guiana, and Peru, between the early 1980s and 2021, and together they present data for 3649 cases of histoplasmosis associated with HIV [37,38,50,51,52,53,54,55,56,57,58,59,60,61,62,63,64,65,66,67,68,69,70,71,72,73,74,75,76,77,78,79,80,81]. Brazil was the country with the most publications, n = 14, and cases, n = 1106 (30%). Studies from Guatemala presented data from 710 cases, including 561 cases reported in the last decade (79% of cases). Colombia and French Guiana presented similar numbers of patients, n = 510 and n = 506, respectively (Table 2) [37,38,50,51,52,53,54,55,56,57,58,59,60,61,62,63,64,65,66,67,68,69,70,71,72,73,74,75,76,77,78,79,80,81]. In most of these reports, the cases were diagnosed by conventional laboratory tests, microscopy/histopathology, and culture (26 of 35 studies; 74%) [37,38,50,52,55,56,57,58,59,60,61,62,63,64,65,66,67,71,72,73,74,76,77,78,79,80]. Studies that used *Histoplasma* Ag testing in urine, n = 9, were mostly conducted after 2014 [51,53,54,68,69,70,74,75,81]. These studies described 1169 of the 3649 histoplasmosis cases reported (32%). In addition, the studies involving the use of Ag testing were characterized by increasing case detection, especially when data were compared with prior statistics of cases diagnosed by a conventional test such as microscopy and culture, increasing the diagnostic yield, in some reports doubling the number of diagnosed cases, or representing the first cases reporting in the country literature [51,54,70,74].

Co-infections were common in 20 of 35 studies, and co-infection rates ranged from 14% to 100%. Histoplasmosis-TB co-infection was also common, reported in 14 studies, with frequency ranging from 18% to 65% of patients with multiple infections (Table 2) [37,38,51,52,53,55,57,58,60,63,64,68,69,70,72,73,75,76,77,79,80,81]

These publications reported variable mortality rates, between 6% to 57% [37,38,50,51,52,53,55,56,57,58,59,60,61,62,63,64,65,66,67,68,69,70,72,73,75,76,77,78,79,80,81]. Two studies that tracked patient mortality over time concluded that a decrease in mortality was observed. The first study from French Guiana described 34 years of experience diagnosing histoplasmosis. In this study, 349 histoplasmosis cases were analyzed, and a crude mortality of 41% over the three decades was observed. In addition, this study analyzed 30-day mortality in four periods: before 1998; 1999 to 2003; 2004 to 2009; and 2010 to 2014. Before 1998, mortality was 38%. From 1999 to 2003, mortality decreased to 17%, probably because of the introduction of ART. In the third and fourth periods, the mortality declines continued to 9% and 6%, respectively. Authors from French Guiana concluded that this decreasing trend was due to progress in adequate HIV care and an increase in awareness and exhaustive laboratory testing for histoplasmosis in French Guiana over the last two decades [76]. The second report, from Guatemala, reported on the implementation of a screening program. During this program, mortality among newly diagnosed HIV patients decreased from 33% in 2017 to 21% in 2019 [51]. This program assessed patients at risk, regardless of the presence of symptoms, and testing was mostly conducted using *Histoplasma* Ag detection in urine [51].

In addition, two more studies have shown how the use of urinary *Histoplasma* Ag detection impacted patients’ mortality. The first, a multicenter study from Guatemala and El Salvador, reported a lower 30-day mortality in patients who were tested for *Histoplasma* Ag, regardless of positive or negative results. Mortality was 13% among patients who underwent testing compared with 75% in patients who were not tested [53]. Another multicenter study from Brazil also showed a benefit of Ag testing, with a 14% mortality rate in individuals tested with the Ag detection assay compared to 27% in those who underwent conventional testing [70].

Several studies identified factors that were associated with mortality in PLHIV with histoplasmosis. These factors include: creatinine > 2.1 mg/dL, albumin concentration < 3.5 g/dL, dyspnea, platelet count <100,000 cell-per-mm3, lactate dehydrogenase concentration elevated to twice the normal value, hemoglobin < 8 g/dL (OR = 3.8; 95% CI 1.4–10.5), AST 2.5 times increased from the limit of normal, and acute renal failure [82,83,84]. Another factor that was found to be associated with poor outcomes was the presence of multiple infections [37,85]. In contrast, ART was found to be a protective factor for severe disease and mortality [72,83]. Treatment relapse was associated with nonadherence to antifungal treatment, central nervous system (CNS) histoplasmosis, and *Histoplasma* antigenuria above 2.0 ng/mL at 1-year follow-up [86].

## 6. Conclusions and Future Perspectives

In recent years, the concept of histoplasmosis as a disease geographically limited to the Americas has been changing, and due to the development of novel diagnostics assays, and increasing disease awareness, a new concept of histoplasmosis as a global disease is becoming more popular [42,45,87,88,89]. Currently, most histoplasmosis cases are reported from the American continent, where it was estimated that in PLHIV with advanced disease, up to 15,600 new cases and 4500 deaths occurred in 2012 [26]. In some countries in Latin America, disease burden estimates are becoming more accurate, likely resulting from the implementation of strategies for improved detection of histoplasmosis in PLHIV [51,53,54,70,74]. Nevertheless, mortality associated with histoplasmosis remains high due to delayed diagnosis of histoplasmosis. Delayed diagnosis may be due to low clinical suspicion of histoplasmosis and limited access to testing [90,91,92].

The availability of rapid testing for histoplasmosis is still limited. Recent publications indicated that *Histoplasma* Ag detection testing and PCR testing were available in approximately 65% of Latin American countries and territories. Common characteristics of laboratories that reported performing histoplasmosis Ag/PCR testing included being specialized and located in major cities [93,94]. Our review indicates that the availability of novel commercial tests for detection of *Histoplasma* Ag detection is having a beneficial effect on case detection and reduction of mortality [44,51,54,70,74,95,96].

Aided by other initiatives, such as the publication of the first WHO global guidelines for the diagnosis and treatment of histoplasmosis in PLHIV, the burden of histoplasmosis and associated mortality may be reduced further. To achieve this, additional investment in laboratory capacity and expertise in fungal diagnosis and clinical management will be needed. The development of more rapid diagnostic testing that can be performed at ‘bed-side’ will provide a quicker diagnosis and initiation of treatment. It is, therefore, essential to have greater access to effective antifungal drugs, such as liposomal amphotericin B and itraconazole oral solution. Current clinical management recommendations are supported by evidence from clinical trials that were conducted in the 1990s and early 2000s. New clinical trials are needed that evaluate shorter courses of antifungal treatment, investigate the efficacy of new generation antifungals, and determine management strategies for patients who suffer from co-infections, especially TB. For PLHIV, these and future advancements in the diagnosis and treatment of histoplasmosis should further result in reduction of mortality and enhanced quality of life.

## Figures and Tables

**Table 1:** Summary of meta-analysis for the analytical performance of assays for the diagnosis of histoplasmosis in PLHIV.

Assay	Sensitivity % (95% CI)	Specificity % (95% CI)	Accuracy % (95% CI)
**Culture**	77 (72–81)	ND	ND
**Antibody detection assays**	58 (53–62)	100 (99–100)	89 (87–91)
**Antigen detection assays**	95 (94–97)	97 (97–98)	95 (94–96)
**Molecular assays**	95 (89–100)	99 (96–100)	96 (94–99)

(95%CI) 95% confidence interval. Adapted from reference [47].

**Table 2:** Analysis of case series of histoplasmosis associated with HIV in Latin America.

Location (Reference)	Period	Study Type		Study Population and Principal Findings (%) Histoplasmosis Frequency	Co-Infections and TB Co-Infection	Mortality
**Central America**						
Guatemala, Guatemala City [50]	2005–2007	Prospective cohort study	No	48 histoplasmosis in 217 patients analyzed (22%)	ND	19/48 (40%)
Guatemala, Guatemala City [37]	2005–2009	Prospective cohort study	No	101 histoplasmosis in 263 patients analyzed (38%)	30% - TB: 25%	44%
Guatemala 13 HIV clinics across Guatemala [51]	2017–2019	Prospective cohort study	Yes	473 histoplasmosis in 6366 patients analyzed (7%)	16% - TB: 57%	Mortality among those who were newly HIV diagnosed showed a decrease at 180 days from 33% in 2017 to 21% in 2019.
Panama, Panama City [52]	1997–2003	Retrospective	No	182 histoplasmosis in 2379 patients analyzed (8%) (104 included for study analysis)	27/104 (26%) - TB: 16/27 (59%)	13/104 (13%)
Guatemala, Guatemala City El Salvador, San Salvador [53]	2012–2014	Multicenter laboratory surveillance	Yes	96 histoplasmosis	44% - TB: 18%	Overall mortality: 18% - 14% in diagnose by Ag testing - 75% in non-diagnosed by Ag testing
- Panama, Panama City - Honduras, Tegucigalpa, and San Pedro de Sula - Nicaragua, Managua [54]	2016–2018	Multicenter laboratory surveillance	Yes	269 histoplasmosis in 1343 patients analyzed (20%) - Panama: 201/857 (23%) - Honduras: 34/106 (32%) - Nicaragua: 34/380 (9%)	ND	ND
**South America**						
Argentina, Buenos Aires [55]	2009–2014	Retrospective	No	171 histoplasmosis	70/171 (41%) - TB: 20/70 (29%)	34/171 (20%)
Argentina, Buenos Aires [56]	2010–2021	Retrospective	No	80 histoplasmosis	ND	26%
Argentina, Buenos Aires [57]	2011–2017	Retrospective	No	37 histoplasmosis	14%	15%
Brazil, Campo Grande [58]	2011–2016	Retrospective	No	23 histoplasmosis	- *Pneumocystis* and TB: 26% - Neuro-toxoplasmosis and bacterial pneumonia: 17% - Visceral leishmaniasis and cryptococcosis: 9% - Syphilis: 4%	57%
Brazil, Ceará State [59]	1995–2004	Retrospective	No	164 histoplasmosis in 378 patients analyzed (43%)	ND	52/164 (32%) - Mortality on ART: 4% - Mortality on non-ART: 28%
Brazil, Ceará State [60]	1999–2005	Retrospective	No	191 histoplasmosis - 134 cases analyzed	ND	44/134 (33%)
Brazil, Ceará State [61]	2012–2013	Cross-sectional study	No	489 buffy coats of 361 patients. Laboratory results: - First culture (proven cases): 61/361 - Sequential culture: 22% sensitivity. - First buffy coat: 19/361 - Sequential buffy coat: 32% sensitivity.	ND	18%
Brazil, Ceará State [60]	2006–2010	Retrospective	No	208 histoplasmosis	ND	88/208 (42%)
Brazil, Goiânia [62]	2003–2014	Retrospective	No	166 histoplasmosis in 2.285 patients analyzed (7%)	ND	88/166 (53%)
Brazil, Manaus [63]	2014–2015	Autopsy analysis	No	14 HIV-Histoplasmosis in 37 HIV-patients analyzed (38%)	8/14 (57%)	8/37 (22%)
Brazil, Mato Grosso do Sul [64]	1998–2005	Retrospective	No	30 histoplasmosis	14/30 (47%)	12/30 (40%)
Brazil, Sao Paulo [65]	2001	Retrospective	No	12 histoplasmosis in 90 patients analyzed (13%) (7.1 cases/1.000 hospitalizations per year)	ND	33%
Brazil, Espírito Santo State [66]	1999–2001	Retrospective	No	12 histoplasmosis in 571 patients analyzed (2%)	ND	2/12 (17%)
Brazil, Uberaba, MG [67]	1992–2005	Retrospective	No	57 histoplasmosis	ND	18/57 (32%)
Brazil, Porto Alegre [68]	2014–2015	Prospective cohort study	Yes	8 proven histoplasmosis by culture (10%) 13 probable by commercial Ag test (17%) 14 probable by in-house Ag test (18%)	25% of TB co-infection in proven histoplasmosis cases	25% in proven histoplasmosis cases
Brazil, Rio Grande [69]	2010–2019	Retrospective	Period 1: No Period 2: Yes	Overall: 31 histoplasmosis - Period 1: 15 histoplasmosis - (8 cases per 1.000 hospitalizations) - Period 2: 16 histoplasmosis - (24 cases per 1.000 hospitalizations)	28/31 (90%) - TB: 9/28 (32%)	11/31 (35%)
Brazil 14 hospitals in the states of: Rio Grande do Norte Bahia Goiás Ceará Rio Grande do Sul Sao Paulo [70]	2016–2018	Prospective cohort study	Yes	123 histoplasmosis in 570 patients analyzed (22%). By state: - Rio Grande do Norte: 13/29 (45%) - Bahia: 4/9 (44%) - Goiás: 50/126 (40%) - Ceará: 23/62 (37%) - Rio Grande do Sul: 26/264 (10%) - Sao Paulo: 7/80 (9%) Urinary antigen detection increased the diagnostic yield in 54%	Coinfection with: - CMV: n = 25 (20%) - TB: n = 19 (15%) - *P. jirovecii*: n = 14 (11%)	Overall, 30-day mortality was 22% - 14% in patients with antigen-based diagnosis (6/42). - 27% in patients with conventional-based diagnosis (21/78)
Colombia, 20 states [71]	1992–2008	Voluntary survey	No	280 histoplasmosis	ND	ND
Colombia, Medellin [72]	1979–2001	Retrospective	No	30 histoplasmosis	21/30 (70%)	7/30 (23%)
Colombia, Medellin [38,73]	2008–2011	Prospective cohort study	No	45 histoplasmosis in 45 patients analyzed (51%)	23/45 (51%) - TB was the most common co-infection (16/23; 70%)	8/45 (18%)
Colombia, 17 states [74]	2009–2012	Prospective cohort study	Yes	105 histoplasmosis in 463 patients analyzed (23%). Implementation of a diagnostic program increased histoplasmosis detection.	ND	ND
Colombia, Pereira [75]	2014–2019	Retrospective	Yes	50 histoplasmosis in 172 patients analyzed (29%)	34/50 (68%) - TB: 22/34 (65%)	14/50 (28%)
French Guiana - Cayenne - Kourou - Saint Laurent du Maroni [76]	1981–2014	Retrospective, multicentric study	No	349 histoplasmosis	137/349 (39%) - TB: 18/137 (13%)	Crude 144/349 (41%) - 30-days: 50/349 (14%) Changes on 30-days mortality among time: - Before 1998: 18/47 (38%) - 1999–2003: 17/100 (17%) - 2004–2009: 10/111 (9%) - 2010–2014: 5/91 (6%)
French Guiana, Saint Laurent du Maroni [77]	2008–2010	Retrospective	No	24 histoplasmosis in 67 patients analyzed (36%)	33%	3/24 (13%)
French Guiana, Cayenne [78]	2009–2018	Retrospective	No	133 histoplasmosis in 227 patients analyzed (59%)	ND	12%
Peru, Lima [79]	1996–2014	Retrospective	No	27 histoplasmosis	23/27 (85%) - TB: 3/23 (13%)	6/27 (22%)
Peru, Lima [80]	2000–2019	Retrospective	No	43 histoplasmosis	TB: 5/43 (12%)	7/43 (16%)

## Data Availability

The data that support the findings of this study are available from the corresponding author upon reasonable request.
